# Mitochondrial movement in pancreatic alpha cells requires Miro2 and is regulated by glucose

**DOI:** 10.1016/j.jbc.2025.110984

**Published:** 2025-11-26

**Authors:** Maia H. Ekstrand, Sameena Nawaz, Anne Clark, Benoit Hastoy, Jakob G. Knudsen

**Affiliations:** 1 Section for Cell Biology and Physiology, Department of Biology, University of Copenhagen, Copenhagen, Denmark; 2 Oxford Centre for Diabetes, Endocrinology and Metabolism (OCDEM), Churchill Hospital, University of Oxford, Oxford, United Kingdom

**Keywords:** glucagon, mitochondria, mitochondrial transport, cell metabolism, insulin, alpha cells, beta cells

## Abstract

Under normal physiological conditions, glucagon is released from pancreatic alpha cells to elevate circulating glucose levels in response to hypoglycemia. In patients with type 2 diabetes, glucagon secretion is dysregulated, but the underlying mechanisms remain unclear. Several hypotheses have been suggested to explain the coupling of blood glucose sensing to electrical activity and glucagon secretion from alpha cells. Here, we show that glucose rapidly regulates mitochondrial motility and localization in alpha cells. Under conditions of low glucose, mitochondria are arrested in positions further from the nucleus, correlating with increased ATP/ADP in the sub-plasma membrane space. We also find that knockdown (KD) of Mitochondrial Rho GTPase 2 (Miro2), but not Miro1, reduces mitochondrial motility in alpha cells and impairs glucose-induced inhibition of glucagon secretion without effects on insulin secretion or mitochondrial motility in non-alpha islet cells. These findings highlight the significance of mitochondrial motility for alpha cell function and reveal fundamental differences between alpha and beta cells.

Tight control of glucagon secretion is required to maintain blood glucose levels within the narrow range of euglycemia (4–5 mM) (1). In patients with type 2 diabetes (T2D), pancreatic alpha cells lose the ability to respond to changes in the circulating glucose levels and glucagon secretion becomes dysregulated by unknown mechanisms (2, 3, 4). Under normal physiological conditions, glucagon release is controlled by both intrinsic and paracrine (intra-islet) mechanisms to respond to circulating blood glucose levels. Several hypotheses have been suggested to explain the mechanisms underlying the intrinsic effects of glucose on glucagon secretion (5, 6, 7, 8). Nevertheless, stimulation of glucagon secretion is ultimately thought to be determined by opening of voltage gated sodium channels and increases in local Ca^2+^ concentrations to depolarize the plasma membrane and facilitate exocytosis of glucagon-containing granules (9, 10, 11, 12). Although the direct coupling of glucose sensing to electrical activity in alpha cells remains debated, oxidative phosphorylation (OXPHOS) and intracellular ATP levels are elevated and sustain glucagon release when extracellular glucose levels are low (6, 13, 14, 15). In other exocytotic cell types, intracellular gradients of OXPHOS and ATP-production are known to play a central role in the establishment of nucleotide-microdomains and vesicle release (16, 17, 18). This correlates with mitochondrial movement and accumulation of mitochondria in distinct cellular compartments to supply ATP for sites of high energy demand (17, 19). The mechanism is well studied in neurons which, due to their asymmetric shape, require mitochondrial mobilization. However, intracellular microdomains of elevated OXPHOS and NAD(P)H have also been reported in more spherical cell types, including pancreatic alpha cells (14, 20, 21). Combined with observations that mitochondrial positioning is important for the buffering of local cytosolic Ca^2+^ concentrations near the plasma membrane (22, 23, 24), this has prompted different hypotheses for the importance of cortical mitochondria in the regulation of pancreatic hormone secretion (25, 26). Here we explore the connection between glucose-regulated mitochondrial motility, localization, and cellular metabolism in pancreatic alpha cells.

## Results

### Mitochondrial motility in alpha cells is acutely suppressed in low glucose

**Figure 1:**
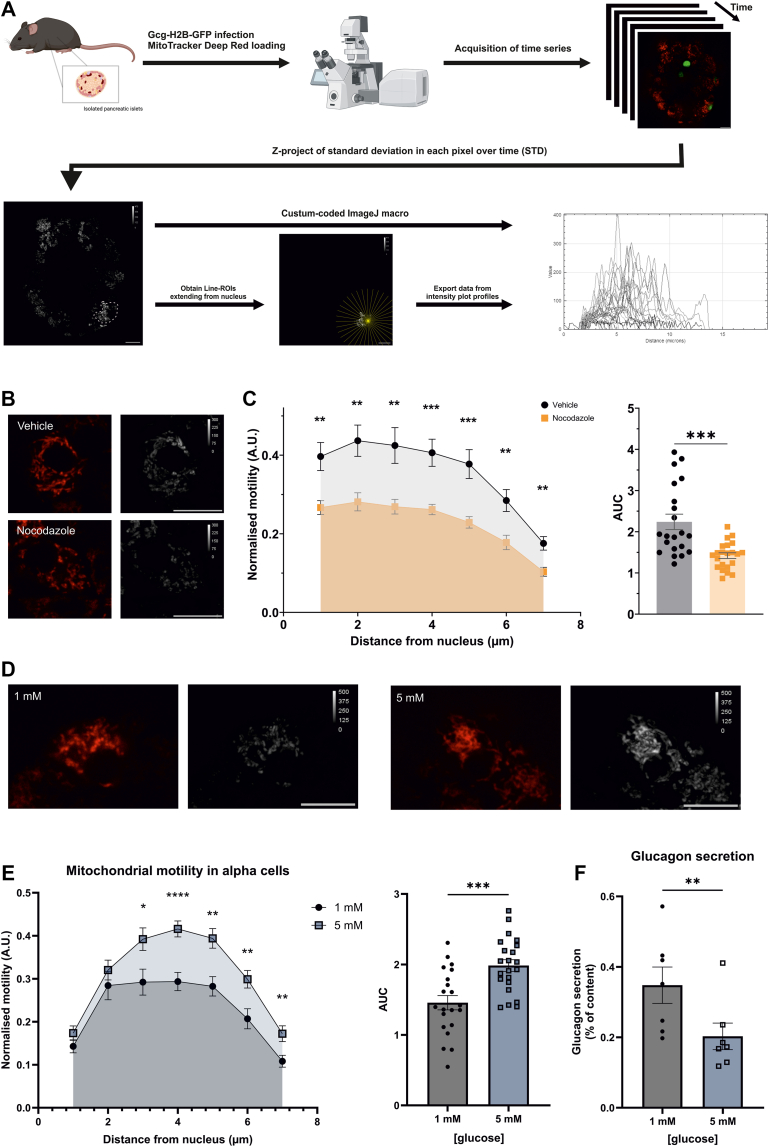
**Mitochondrial motility is acutely suppressed by low glucose in pancreatic alpha cells.***A*, schematic of the imaging- and analysis workflow developed to assess mitochondrial motility in whole pancreatic islets. Whole isolated islets are infected with an adenoviral construct delivering histone-bound GFP under control of the glucagon promoter (Gcg-H2B-GFP) for 36–48 h. Islets are then loaded with MitoTracker DeepRed and imaged in short time series. Each time series is collapsed into a z-project of the standard deviation over time and a custom coded ImageJ macro is used to obtain plot profiles of the standard deviation from the nucleus to the cell periphery. *B*, representative images of mitochondrial motility in islet cells treated with 5 μM nocodazole or corresponding vehicle. *C*, quantification of mitochondrial motility in cells from whole isolated islets incubated in 5 μM nocodazole or corresponding vehicle (n = 21–22 cells from 3 mice). *D*, representative images of mitochondrial motility in alpha cells from whole islets incubated in 1 mM or 5 mM glucose for 15 min. *E*, quantification of mitochondrial motility in alpha cells from whole isolated islets incubated in 1 mM or 5 mM glucose (n = 21–22 alpha cells from 6 mice). *F*, glucagon secretion measurements from whole islets incubated in parallel in glucose as indicated (n = 7). ∗*p* < 0.05, ∗∗*p* < 0.005, ∗∗∗*p* < 0.001, ∗∗∗∗*p* > 0.0001 with unpaired *t* test or two-way ANOVA. Scalebars are 10 μm.

To test whether glucose regulated mitochondrial motility in alpha cells, we first established complementary imaging tools to reliably quantify mitochondrial motility from confocal live cell recordings of whole isolated mouse islets. Existing mitochondrial tracking algorithms, optimised for essentially two-dimensional cells like monolayer cultures, are unsuitable for primary islet cells, where mitochondria fluctuate in and out of the imaging plane. Mitochondria were visualised using MitoTracker Deep Red, and motility was quantified as the standard deviation of signal (Figs. 1*A* and S1*A*). Standardized units of motility were obtained by normalizing to the maximal motility in each cell. Mitochondrial motility was analyzed in cytosolic fractions of 7 μm, measured from the periphery of the nucleus to the plasma membrane. These criteria were based on preliminary analyses evaluating the frequency distribution of lines of different lengths (corresponding to cytosolic fractions) and intensity heat plots from a large data set of control cells, revealing cytosolic fractions of 7 μm to be the most suitable compromise between observation frequency and mitochondrial occurrence (Fig. S1, *B*–*D*). Since the MitoTracker DeepRed signal intensity is sensitive to the mitochondrial membrane potential, we controlled whether it could interfere with the assessment of mitochondrial motility under the conditions tested here. Irrespective of the glucose level, the standard deviation of the signal did not correlate with the average intensity of signal, indicating that differences in standard deviation are minimally influenced by variation of membrane potential and/or quenching of the signal (Fig. S1*E*). We validated the responsiveness of the method by measuring the motility of mitochondria in islets treated with the microtubule destabilizer, nocodazole, serving as a negative control (Fig. 1*B*). Compared to vehicle-treated islet cells, we confirmed suppressed mitochondrial motility throughout the cytosolic fraction in nocodazole-treated islets (Fig. 1*C*).

To identify alpha cells, whole isolated islets were infected with an adenoviral construct expressing nuclear localized GFP under the preproglucagon promoter (Gcg-H2B-GFP) (Fig. 1*A*) (6). After 15 min in 5 mM glucose, mitochondrial motility was increased by 25% compared to 1 mM throughout the cytosolic fraction (Fig. 1, *D* and *E*), without changes in the maximal motility measured. This was consistent after incubation in 7 mM glucose (Fig. S2*A*). The increased mitochondrial motility in 5 mM glucose also correlated with reduced glucagon secretion (Fig. 1*F*). This suggests that changes in mitochondrial motility in response to glucose are associated with the regulation of glucagon secretion.

### Mitochondria in alpha cells reposition in response to acute changes in glucose

**Figure 2:**
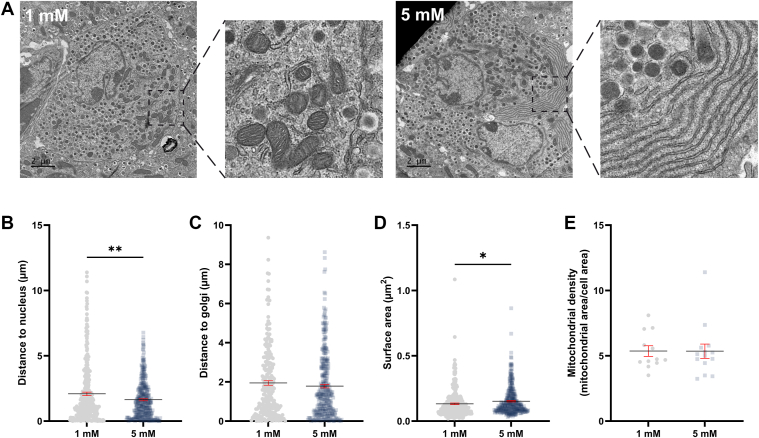
**Mitochondria acutely localize further from the alpha cell nucleus in low glucose.***A*, transmission electron micrographs of alpha cells from whole isolated islets incubated in 1 mM or 5 mM glucose as indicated. *B–C*, the shortest distance from each mitochondrion to the nucleus or Golgi apparatus, respectively. *D*, Mitochondrial surface area. *E*, mitochondrial density measured as the ratio of total mitochondrial area to total cell area (n = 309–346 mitochondria from 12-14 alpha cells). ∗*p* < 0.05, ∗∗*p* < 0.005 with unpaired *t* test.

As mitochondrial motility depends on glucose level, we next investigated whether the reduced motility at low glucose positioned mitochondria in distinct cellular compartments. Whole mouse islets were incubated in 1 mM or 5 mM glucose for 15 min prior to being fixed and processed for transmission electron microscopy (TEM) (Fig. 2*A*). By automatically computing the shortest distance from each mitochondrion to the nucleus and Golgi apparatus, respectively, we found that mitochondria in alpha cells are localized further from the nucleus when incubated in 1 mM glucose compared to 5 mM glucose (Fig. 2*B*) while the orientation to the Golgi apparatus was retained (Fig. 2*C*). Notably, cell size did not differ between the different glucose treatments (Fig. S2*E*). Similarly, aspect ratio (length-to-width ratio), form factor (branching), and circularity of each mitochondrion were comparable between incubation in 1 mM or 5 mM glucose, indicating that mitochondrial morphology was unaffected by short-term changes in glucose concentration (Fig. S2, *B*–*D*). The surface area of individual mitochondria was, however, increased after incubation in 5 mM glucose (Fig. 2*D*), perhaps linked to rapid metabolic adaptation. This was without changes in mitochondrial density (Fig. 2*E*). These findings suggest that mitochondrial halt leads to accumulation of mitochondria further away from the nucleus in low glucose.

### Extracellular glucose regulates ATP gradients in the sub-plasma membrane compartment

**Figure 3:**
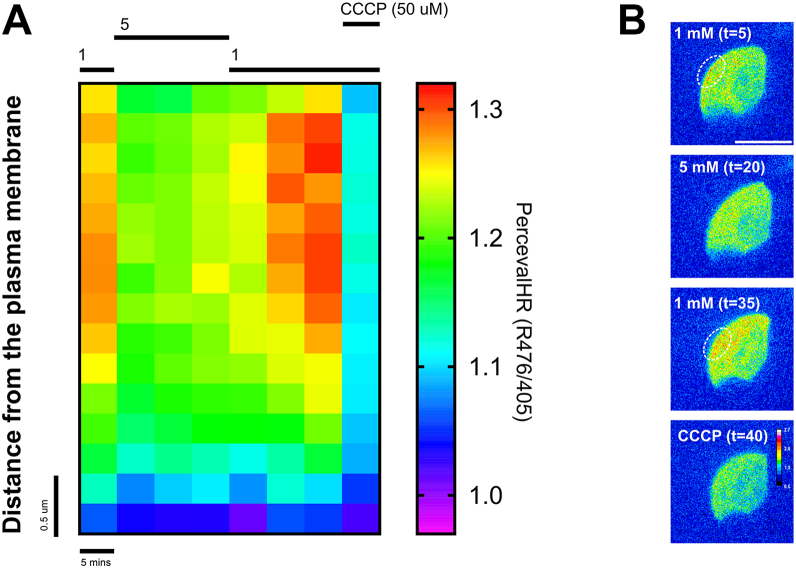
**Glucose regulates the ATP/ADP ratio in the sub-plasma membrane compartment of alpha cells.***A*, ratiometric PercevalHR intensity (R476/405) in the sub-plasma membrane space (from plasma membrane to 4 μm below the plasma membrane) of alpha cells in whole isolated islets perifused with glucose or CCCP (50 μM) as indicated (n = n = 18 cells from 4 mice). *B*, representative images of the ATP/ADP ratio in an alpha cell subjected to glucose or CCCP as indicated, showing the presence of sub-plasma membrane microdomains of high ATP/ADP in low glucose (circled). Scalebar is 10 μm.

To explore whether the glucose-induced change in mitochondrial positioning correlated with changes in intracellular ATP gradients in alpha cells, we performed live cell ATP imaging using an alpha cell-specific version of the ATP/ADP sensor, PercevalHR (6). The intracellular ATP/ADP ratios in alpha cells were higher in 1 mM compared to 5 mM glucose, consistent with earlier reports (6, 13, 14). We next quantified the intracellular ATP gradient in the sub-plasma membrane space (<4 μm below the plasma membrane) in response to 1 mM and 5 mM glucose. An increase in glucose from 1 mM to 5 mM reduced ATP/ADP in the sub-plasma membrane space, and when the glucose concentration was returned to 1 mM, the ATP/ADP ratio gradually increased (Fig. 3*A*). The gradient was entirely diminished upon addition of the mitochondrial uncoupler, carbonyl cyanide m-chlorophenylhydrazone (CCCP) (Fig. 3*A*). The increased ATP/ADP ratio in the sub-plasma membrane space could be ascribed to the accumulation of ATP in secluded microdomains. In 11 out of 18 cells, we observed microdomains of ATP just below the plasma membrane in 1 mM glucose, which partially or fully disappeared after glucose elevation (Fig. 3*B*). These findings suggest that compartmentalized accumulation of ATP could correlate with mitochondrial localization in alpha cells.

### Miro1 is dispensable for mitochondrial motility in alpha cells

**Figure 4:**
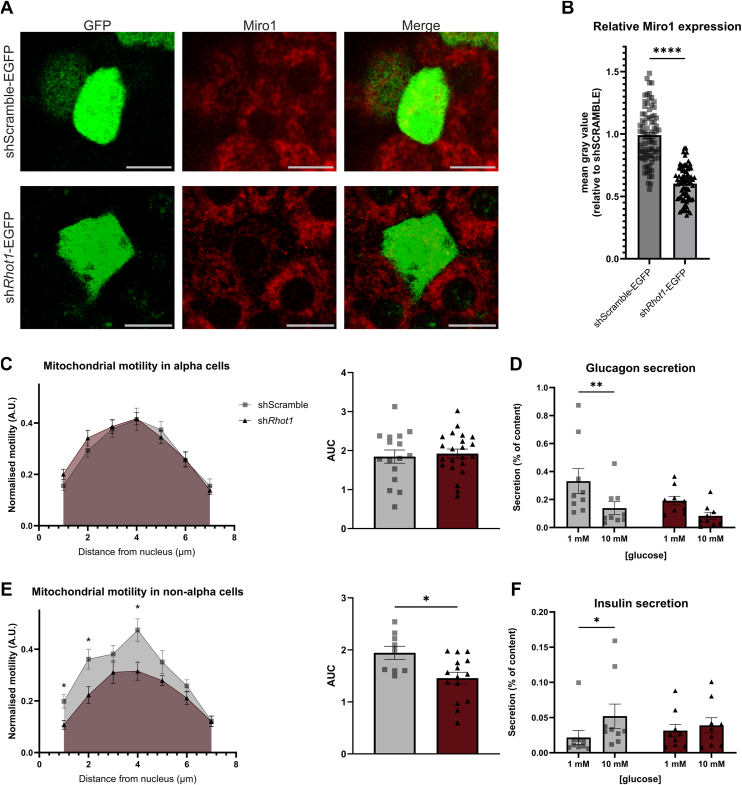
**Mitochondrial motility in alpha cells is unchanged with reduced expression of Miro1.***A*, immunofluorescence stainings of Miro1 and GFP (co-stained with glucagon to identify alpha cells) in whole isolated islets infected with shScramble-EGFP or sh*Rhot11*-EGFP. *B*, Miro1 KD efficiency assessed by immunofluorescence stainings and normalized to shScramble (n = 78 cells from 3 mice, hereof 64–65% alpha cells). *C* and *E*, mitochondrial motility in alpha cells (*C*) and non-alpha cells (*E*) infected with shScramble or sh*Rhot1* in 7 mM glucose (n = 9–20 cells from 3 mice). *D*, glucagon secretion and (*F*) insulin secretion from islets infected with shScramble or sh*Rhot1* (n = 9). ∗*p* < 0.05, ∗∗*p* < 0.005, ∗∗∗∗*p* < 0.0001 with unpaired *t* test or two-way ANOVA. Scalebar is 10 μm.

In neurons and several other cell types, the paralogues Miro1 and Miro2 (encoded by the genes *Rhot1* and *Rhot2)* are a part of the mitochondrial trafficking complex embedded in the outer mitochondrial membrane (27). While less is known about Miro2, the conformation of Miro1 is sensitive to changes in intracellular Ca^2+^ (28), and increases can lead to dissociation of the mitochondria from the trafficking complex, halting mitochondrial motility (29). We therefore speculated that mitochondrial motility and secretory functions in alpha cells may depend on Miro1 or Miro2. Immunofluorescence staining revealed expression of both Miro1 and Miro2 in alpha and beta cells from both mouse and human islets (Fig. S3), consistent with previously published transcriptomics data (30, 31). We first tested whether the glucose-induced regulation of mitochondrial motility and localization in alpha cells was Miro1 dependent. Lentiviral delivery of a construct expressing a short hairpin RNA (shRNA) against Miro1 was co-expressed with EGFP to identify infected cells (sh*Rhot1*-EGFP) (Fig. 4*A*). Using measurements of immunofluorescence, a KD efficiency of approximately 40% was observed in cells expressing the sh*Rhot1*-EGFP construct compared to cells expressing an shScramble control (shScramble-EGFP) (Fig. 4, *A* and *B*).

To determine the effect of Miro 1 KD on mitochondrial motility in alpha cells, islets were double-infected with shScramble/*Rhot1* and Gcg-H2B-GFP and mitochondria were visualised with MitoTracker. Despite mitochondrial motility being unaffected by Miro1 KD in alpha cells (Fig. 4*C*), glucose-induced inhibition of glucagon secretion was compromised (Fig. 4*D*). In contrast, non-alpha cells displayed reduced relative mitochondrial motility (with no changes in the maximal motility measured) and impaired glucose-stimulated insulin secretion (GSIS) when Miro1 expression was repressed (Fig. 4, *E* and *F*). These findings are consistent with previous observations, indicating that Miro1 is important for glucose-induced regulation of hormone secretion from beta cells (32, 33). The effects on glucagon secretion suggest either that Miro1 plays a role in the regulation of glucagon secretion independently of mitochondrial motility, or that Miro1 KD may have indirect effects, possibly through paracrine mechanisms. In support of this latter hypothesis, reduced expression of Miro1 altered mitochondrial localization and size in beta cells but not in alpha cells (Fig. S4). These findings underline a central role for Miro1 in the regulation of mitochondrial motility, localization, and size in beta cells but not in alpha cells.

### Miro2 regulates mitochondrial motility in alpha cells

**Figure 5:**
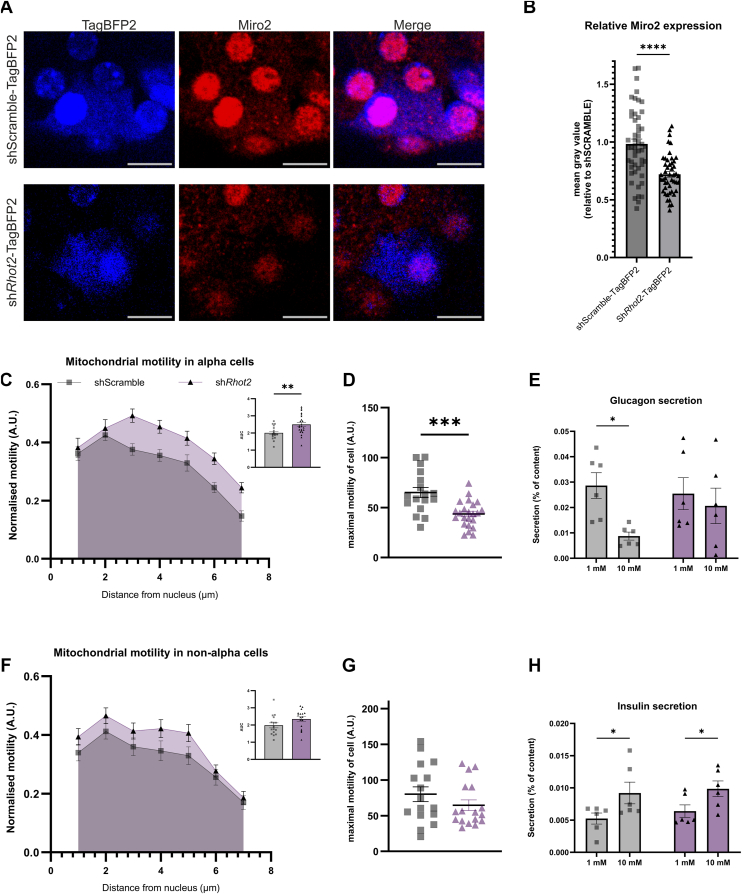
**Mitochondrial motility is impaired in alpha cells with reduced Miro2 expression.***A*, immunofluorescence stainings of Miro2 and expression of TagBFP2 (co-stained with glucagon to identify alpha cells) in whole isolated islets infected with shScramble-TagBFP2 or sh*Rhot2*-TagBFP2. *B*, Miro2 KD efficiency assessed by immunofluorescence stainings and normalized to shSCRAMBLE (n = 47–50 cells from 3 mice, hereof 43–78% alpha cells). *C*, *D*, *F*, and *G*, normalized mitochondrial motility and maximal motility in alpha cells (*C* and *D*) and non-alpha cells (*F* and *G*) infected with shScramble or sh*Rhot2* in 7 mM glucose (n = 16–23 cells from 5 mice). *E*, glucagon secretion and (*H*) insulin secretion from islets infected with shScramble or sh*Rhot2* (n = 6). ∗*p* < 0.05, ∗∗*p* < 0.005, ∗∗∗*p* < 0.001, ∗∗∗∗*p* < 0.0001 with unpaired *t* test or two-way ANOVA. Scalebar is 10 μm.

Based on our observations that Miro1 is unlikely to be a key regulator of mitochondrial motility in alpha cells, we next examined the related paralogue, Miro2. Islets were infected with a *Rhot2* or Scramble shRNA construct fused to TagBFP2 (sh*Rhot2*/Scramble-TagBFP2). This resulted in approximately 30% reduction in Miro2 content (Fig. 5, *A* and *B*). In alpha cells with reduced Miro2 expression, the maximal mitochondrial motility was reduced, resulting in an apparent increase in normalized motility compared to the shScramble control (Fig. 5, *C* and *D*). This indicates that while Miro2 KD may lead to higher relative motility, it reduced the overall motile capacity in alpha cells. No effect was seen on mitochondrial motility in non-alpha cells with reduced expression of Miro2 (Fig. 5, *F* and *G*). Accordingly, glucose-induced inhibition of glucagon secretion was impaired in Miro2 KD islets (Fig. 5*E*) while GSIS from beta cells was unaffected (Fig. 5*H*). These results point to Miro2 as the main regulator of mitochondrial motility in alpha cells, revealing distinct mechanisms for the regulation of mitochondrial dynamics between alpha and beta cells.

## Discussion

**Figure 6:**
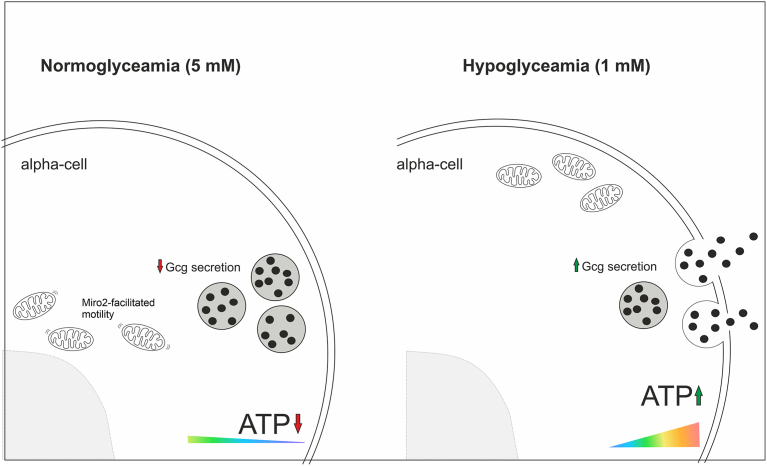
**Representation of the relationship between mitochondrial motility, positioning, and intracellular ATP in alpha cells.** Visual summary of the present findings showing Miro2-facilitated mitochondrial motility and peri-nuclear mitochondria in alpha cells at normoglyceamia, concurrent with low sub-plasma membrane ATP and low glucagon secretion. At hypoglyceamia, mitochondrial motility is suppressed, and mitochondria accumulate further from the nucleus, correlated with high sub-plasma membrane ATP and high glucagon secretion.

Here, we present a new method for quantifying mitochondrial motility in whole pancreatic islets, where mitochondria often disappear and reappear in the two-dimensional plane of imaging, preventing the employment of existing tracking algorithms. Using chemical inhibition and knockdown of mitochondrial Rho GTPases, we confirmed that the method reliably reports mitochondrial motility in primary islets. Using this method, we demonstrate that in pancreatic alpha cells, mitochondrial motility is acutely regulated by glucose and under hypoglycemic conditions, mitochondria are halted in locations that are further away from the nucleus, possibly supporting elevated sub-plasma membrane ATP under conditions of glucagon secretion (Fig. 6).

While the underlying molecular mechanisms of glucose-induced changes in glucagon secretion and intracellular ATP in alpha cells are still debated (7, 34, 35), it appears clear that changing the level of ATP is crucial for the regulation of glucagon release (34, 35). Previous measurements of intracellular ATP, with one exception (36), reflect whole cytosolic changes in ATP (35, 37, 38, 39). The data presented here suggests that it is not only the total cytosolic concentration of ATP that changes with glucose, but that sub-plasma membrane microdomains with high ATP/ADP occur at low glucose levels, and that the ATP/ADP ratio in the sub-plasma membrane space is reduced acutely with increases in extracellular glucose. These spatiotemporal dynamics match the dynamics observed in mitochondrial motility and localization, where low glucose cause mitochondrial movement to slow down closer to the cell periphery. These findings suggest that local microdomains of ATP near the plasma membrane supply energy for electrical activity and exocytosis of glucagon granules at low glucose. Whether the mitochondria are in close proximity to the plasma membrane, as suggested in beta cells (18, 26), is unclear as the two-dimensionality of the data reported here prevents us from accurately measuring the absolute proximity of the mitochondria to the plasma membrane. The reason being that two-dimensional images will typically reveal the genuine projection from a mitochondrion to the nearest point on a compact sphere like the nucleus, while the shortest projection to the plasma membrane, a much larger sphere, often go beyond the plane that is visible in the two-dimensional electron micrograph.

In beta cells, Miro1 has been shown to play an important role in the maintenance of mitochondrial structure and insulin release (32, 33). This is supported by our findings where loss of Miro1 leads to lower mitochondrial motility in non-alpha cells. In contrast, Miro2 KD directly reduced mitochondrial motility only in alpha cells, coinciding with disrupted regulation of glucose induced inhibition of glucagon secretion. This demonstrates for the first time fundamental differences in the regulation of mitochondrial dynamics between alpha and beta cells. While some functional redundancy between Miro1 and -2 cannot be ruled out, these findings point to distinct cell type-specific roles, consistent with previous studies showing that the paralogs vary in their relative importance across cell types (40). Suppressing the expression of Miro1 in pancreatic islets compromised the regulation of glucagon secretion without engaging changes in alpha cell mitochondrial motility. While this effect could originate from an indirect mechanism caused by the impact of Miro1 KD on other islet cells, Miro1 may also directly regulate alpha cell secretion *via* mechanisms unrelated to mitochondrial motility. Although Miro1 KD had little effect on mitochondrial dynamics and positioning in alpha cells, we did observe a reduction in cell size, causing mitochondria to display shorter distances to the Golgi apparatus (this effect disappeared when controlling for cell size). Reduced cell size can be ascribed to increased endocytosis or decreased exocytosis activity, possibly linking this observation to the reduced glucagon secretion from islets with reduced expression of Miro1. However, the intrinsic role of Miro1 in alpha cells may extend beyond mitochondrial motility to include regulation of the cell cycle (41) or mitochondrial quality control and consequential cell stress (42) as observed in other cell types, which could also underlie the observed reduction in alpha cell size following Miro1 KD.

Mitochondrial size and architecture are generally linked to metabolic adaptations and nutrient utilization in other cell types (43). Specifically, increased curvature of the mitochondrial membrane is associated with improved utilization of long-chain fatty acids and increased fatty acid oxidation (FAO) caused by lower sensitivity of carnitine palmitoyl transferase 1 (CPT1) to the inhibitory effect of malonyl-CoA in the outer membrane of smaller mitochondria (44). Considering this, the smaller mitochondria observed in alpha cells after incubation in 1 mM glucose may represent a bioenergetic adaptation to favor the utilization and oxidation of fatty acids. This interpretation is consistent with previous studies, demonstrating compromised ATP production and impaired glucagon secretion from alpha cells where CPT1 has been inhibited (6, 15).

In conclusion, our findings underline the prompt mitochondrial dynamics in islet cells in correlation to changes in nutrient availability and highlight an important role for Miro2 in facilitating mitochondrial adaptations to support alpha cell function.

## Experimental procedures

### Animals

Female C57BL/6Nrj mice were housed in same-sex littermate groups of 2 to 10 mice and used at 12 to 20 weeks of age. Mice were kept in a temperature- and humidity-controlled room and on a 12 h light/dark cycle with ad libitum access to water and a regular chow diet. All experiments were performed in accordance with ethical approval from the Danish animal inspectorate.

### Human islets

All work with human material was approved by the National Committee for the Ethical Approval of Research, Denmark and abides by the Declaration of Helsinki principles. Human islets were obtained from the ADI Islet Core Laboratory at the University of Alberta, Canada. The donor was non-diabetic (Sex: Male, Age: 56 years, BMI: 23.1, HbA1c: 5.6%). Islets were cultured at 37 °C, 5% CO2 in RPMI 1640 (61870-010) (10% FBS, 1% penicillin/streptomycin).

### Islet isolation

Mice were euthanized by cervical dislocation in the morning, and Liberase (Roche, 332 05401020001) injection was used to inflate and digest the pancreas. Digested pancreata were mechanically disrupted by hand and washed in ice-cold Hanks’ balanced salt solution (HBSS) (Sigma-Aldrich, H6648) containing 0.2% BSA. Subsequently, islets were hand-picked into RPMI 1640 culture medium supplemented with 7 mM glucose, 10% FBS and pen/strep (100 U/ml/100 μg/ml). Isolated islets were cultured at 37 °C, 5% CO_2_ for at least an hour until use in further experiments.

### Live-cell mitochondria imaging

To identify alpha cells, whole isolated islets were transduced for 36-48 h with an adenovirus delivering nuclear localized (H2B) GFP under the preproglucagon promoter (Gcg-H2B-GFP). Infected islets were imaged in a PH3 or PH6 imaging chamber (Warner instruments) assembled with a coverslip and filled with Krebs Ringer Bicarbonate (KRB) solution (115 mM NaCl, 3.5 mM KCl, 2.5 mM CaCl2, 0.5 mM MgSO4, 0.5 mM NaH2PO4, 25 mM NaHCO3, 5 mM HEPES, pH 7.4 at 37 °C) containing 0.36 mM fatty acids (21% palmitate, 45.5% oleic acid and 22.8% linoleic acid) bound to fatty acid free BSA in a 3.6:1 M ratio and glucose as indicated for each experiment. Islets were loaded with 100 to 500 nM MitoTracker Deep Red (644/665 nm) (Invitrogen, M22426) (diluted in KRB with glucose as indicated from a 1 mM stock in dimethyl sulfoxide (DMSO)) for 15 min before being transferred directly onto the coverslip in the chamber. For nocodazole treatment, islets were incubated in culture media with 5 μM nocodazole (or corresponding amount of DMSO for vehicle treated islets) prior to being transferred to 7 mM glucose KRB with MitoTracker and 5 μM nocodazole or vehicle. A 180 μm mesh was mounted on the coverslip to keep the islets in place, and the islets were allowed to settle on the coverslip for 5 min before the recordings were started. Islets were imaged on a Leica SP5X confocal line-scanning microscope using a 63x water objective or a Nikon Ti2 microscope with a Crest X-light V3 spinning disk confocal using a 60x oil objective. The fluorophores were excited using white light lasers at 488 nm (or 476 nm) and 644 nm (or 647 nm) for GFP and MitoTracker, respectively, and fluorescence was recorded at 500 to 550 nm (or 511–520) and 660 to 720 nm (or 663–738). Images with a section thickness of ∼1 μm were captured every 5 s to acquire a time-series of approximately 1.5 min.

### Mitochondrial motility analysis

Mitochondrial motility and distribution were analyzed using original code written for Fiji/ImageJ (available through github). Individual cells in the islet were manually outlined as ROIs and defined as alpha or non-alpha cells based on nuclear GFP fluorescence. We use the standard deviation in MitoTracker signal intensity in each pixel over time as a measure of mitochondrial motility. This was done by collapsing each time series into z-projects of the standard deviation of intensity using Fiji/ImageJ. As a control for bleaching and general fluctuations in the signal (*e.g.* due to sensitivity of the MitoTracker to changes in the mitochondrial membrane potential), the same was done with the average signal over time (data not shown). To obtain relative values for motility in each cell and to account for any inherent background movement in the recordings as well as possible effects of MitoTracker loading, the standard deviation of the signal was normalized to the maximal value observed in each cell. The nucleus was semi-automatically defined by thresholding, and intensity profiles were obtained for 36 line-ROIs extending from the centroid of the nucleus at a 10° angle (θ = 10°). We also tested whether it would give a more detailed output to obtain data from 360 line-ROIs per cell (θ = 1°), however, no major differences were observed (Fig. S1, *F* and *G*). The data output was analyzed by averaging the intensity profiles for all line-ROIs of 7 μm (the length from the nucleus to the cell membrane) within a cell and compared between conditions. Based on preliminary testing and analysis (Fig. S1), these were found to be the most suitable parameters for avoiding variation and contribution from background signal.

### Intracellular ATP/ADP imaging

For imaging of intracellular ATP/ADP, isolated islets were transduced for 36–48 h with an adenovirus delivering the ATP/ADP sensor, PercevalHR, under the preproglucagon promoter. Islets were imaged in a heat-controlled, ultraquiet PH6/JG-23 imaging chamber (Warner Instruments) and were perfused with KRB containing glucose and 50 μM of the mitochondrial uncoupler, CCCP, as indicated in the figure. Before imaging, islets were pre-incubated in the chamber containing KRB with 1 mM glucose for 15 to 20 min. The chamber was fitted on a Nikon Ti2 microscope with a Crest X-light V3 spinning disk confocal. The probe was excited using lasers at both 405 nm and 476 nm at 15% and 5% laser power, respectively, and a 511/520 emission filter. Fluorescence emission was collected through a 40x silicon objective every 2.5 min with a Teledyne Kinetix sCMOS camera and quantified ratiometrically (R476/405). Since the detection of quantifiable, intracellular gradients in PercevalHR signal was heavily dependent on the expression and excitation of the probe, only about 2 to 5 cells per 60 islets had the required expression levels.

### Hormone secretion

Glucagon and insulin secretions were measured from size-matched triplicates of 10 islets for each replicate. Islets were pre-incubated in KRB containing 0.36 mM fatty acids bound to BSA in a 3:1 M ratio and 1 mM glucose for 1 h at 37 °C, 5% CO_2_. Unless otherwise stated, islets were incubated sequentially in first 1 mM glucose, followed by incubation in 10 mM glucose. For parallel hormone secretion experiments, islets were subjected to either 1 mM or 5 mM glucose following pre-incubation. Islets were harvested in cold acid ethanol (23.5% acetic acid, 1.5% hydrochloric acid in ethanol), and all samples were stored at −80 °C until further analysis. Glucagon concentrations in supernatants and contents were measured using a glucagon HTRF assay kit (PerkinElmer, 62CGLPEG). Insulin was measured using an ultra-sensitive insulin HTRF assay kit (PerkinElmer, 62IN2PEG).

### Transmission electron microscopy

Whole mouse islets were stimulated with glucose (as indicated) in KRB with 0.36 mM fatty acids bound to BSA in a 3:1 M ratio for 15 min at 37 °C, 5% CO_2_ and fixed in 2.5% EM grade glutaraldehyde (Sigma-Aldrich, G5882) in phosphate buffer (0.1 M, pH 7.2). Samples were washed in cacodylate buffer (0.1 M, pH = 7.4) and post-fixed in 1% osmium, 1.5% sodium ferricyanide, followed by 2% uranyl acetate. Islets were dehydrated and embedded in Spurr’s resin. Ultra-thin sections were cut onto nickel grids and contrasted with uranyl acetate. Sections were imaged on a Jeol JEM-1400Flash transmission electron microscope with a Gatan OneView 16-megapixel camera accessed through the Centre for Bioimaging at Oxford Brookes University.

TEM images were analyzed using Fiji/ImageJ, and cell type differentiation was performed by visual inspection using cues as secretory granule appearance (determined by two people independent of each other) (45, 46, 47). For each cell, the nucleus, plasma membrane, Golgi, and mitochondria were manually annotated. Sections of cells were selected based on the following selection criteria: the plasma membrane was distinguishable around the whole cell; the cellular section contained a clearly visible nucleus; the section contained one or more mitochondria. Mitochondria were manually defined based on gray value, and both mitochondria with visible cristae and non-visible cristae were selected. Mitochondrial morphology attributes, as well as the shortest distance/projection from each mitochondrion to the nucleus, plasma membrane, and Golgi, were obtained using original code for Fiji/ImageJ. Morphological attributes were calculated as follows: Aspect Ratio (AR) = length/width, Form Factor (FF) = perimeter^2^/(4∗π∗area), Circularity = 4∗π ∗(area/perimeter^2^). Data on the distances from mitochondria to the Golgi were limited to cellular sections where part of the Golgi apparatus was visible. If the Golgi apparatus appeared in several non-connected places in one section of a cell, the projection from each mitochondrion to the closest part of the Golgi apparatus was automatically computed. Cristae were counted only in mitochondria where the whole mitochondrial area contained visible cristae. In islets infected with sh*Rhot1* or shScramble, cells were selected based on the abovementioned criteria regardless of whether the cellular section contained visible virus particles or not.

### shRNA gene silencing

To knock down the expression of *Rhot1* and *Rhot2*, whole isolated islets were transduced with a lentiviral construct containing EGFP and a mix of three different sh*Rhot1* sequences (sh*Rhot1*-EGFP) or TagBFP2 and a mix of three different sh*Rhot2* sequences (sh*Rhot2*-TagBFP2) downstream of a U6 promoter. sh*Rhot1*-EGFP and sh*Rhot2*-TagBFP2 consisted of a 1:1:1 plaque forming units (PFU) mix of viruses containing three different shRNA sequences (*Rhot1* vector IDs: VB221209-1133guv, VB221209-1134gxg, and VB221209-1132vzy. *Rhot2* vector IDs: VB250220-1085cmw, VB250220-1086ypq, and VB250220-1087hkj). shRNA sequences were tested individually but were observed to be most efficient when used together. Control cells were transduced with a similar construct, expressing a scrambled shRNA sequence (shScramble-EGFP or shScramble-TagBFP2). Lentivirus was obtained from VectorBuilder (3LVS (LVshRNA)-C-59040-6). Islets were infected for 48 h with lentivirus to a multiplication of infection (MOI) of 30. Efficiency of KD was quantified from immunofluorescence staining images using a rolling ball radius of 300px to subtract background fluorescence prior to quantification of Miro1 or -2 staining in infected cells.

### Immunofluorescence labeling

Whole isolated islets were fixed in 10% formalin (∼4% PFA) (Sigma-Aldrich, HT501128). Islets were washed in PBS with 2% BSA and permeabilized with 0.1 to 1% Triton X-100. Following permeabilization, islets were blocked in PBS with 2% BSA and incubated in primary antibodies against Miro1 (Sigma ZRB1674), Miro2 (Abcam ab154946), Glucagon (Sigma G2654) or GFP (Abcam ab13970) at 4 °C overnight. The primary antibody was washed off in PBS with 2% BSA before incubation with Alexa Fluor secondary antibodies. Immunofluorescence images of islets were acquired with a Leica SP5X confocal line-scanning microscope using a 63x water objective or with a Nikon Ti2 microscope with a Crest X-light V3 spinning disk confocal using a 60x oil objective.

### Statistical analyses

Results are shown as mean ± standard error of mean (SEM) unless otherwise stated. All statistical analyses were conducted using GraphPad Prism software (version 10). Unpaired or paired Student's *t* test was used for pairwise comparison as indicated in the figure legends. For multiple interactions analysis, one- or two-way analysis of variance (ANOVA) was used with correction for degrees of freedom and followed by Tukey’s or Sídáks *post hoc* tests. Outliers were removed using ± 2SD. *p*-values less than 0.05 were considered significant.

## Data availability

All data required to assess our conclusions are included in the manuscript. Raw images and source data for all figures can be obtained upon reasonable request to the corresponding author (JGK, jgknudsen@bio.ku.dk). All original code is available through the GitHub repository: https://github.com/KnudsenLab/mitochondrial-dynamics-analysis.

## Supporting information

This article contains supporting information.

## Conflict of interest

The authors declare that they do not have any conflicts of interest with the content of this article.
