# Supplementary material for: Mitochondrial movement in pancreatic alpha cells requires Miro2 and is regulated by glucose
Source: J Biol Chem. 2025 Nov 26;302(1):110984. doi: 10.1016/j.jbc.2025.110984 (PMC12774744; doi:10.1016/j.jbc.2025.110984)
Supplement: Supporting Figures [file mmc1.pdf]

Mitochondrial movement in pancreatic alpha cells requires Miro2 and is regulated by glucose

Maia H. Ekstrand[1], Sameena Nawaz[2], Anne Clark[2], Benoit Hastoy[2], Jakob G. Knudsen[1,*]

1, Section for Cell Biology and Physiology, Department of Biology, University of Copenhagen

2, Oxford Centre for Diabetes, Endocrinology and Metabolism (OCDEM), Churchill Hospital, University of Oxford

*Correspondence: jgknudsen@bio.ku.dk

The following material is included:

Figure S1. Establishing a method for analysis of mitochondrial motility in whole pancreatic islets ex vivo

Figure S2. Mitochondrial motility is comparable between 5 mM and 7 mM glucose and mitochondrial morphology and alpha cell size is unchanged after incubation in 1 mM or 5 mM glucose

Figure S3. Miro1 and Miro2 is expressed in both alpha and beta cells in mice and humans

Figure S4: Reduced expression of Miro1 alters mitochondrial localisation and size in beta cells

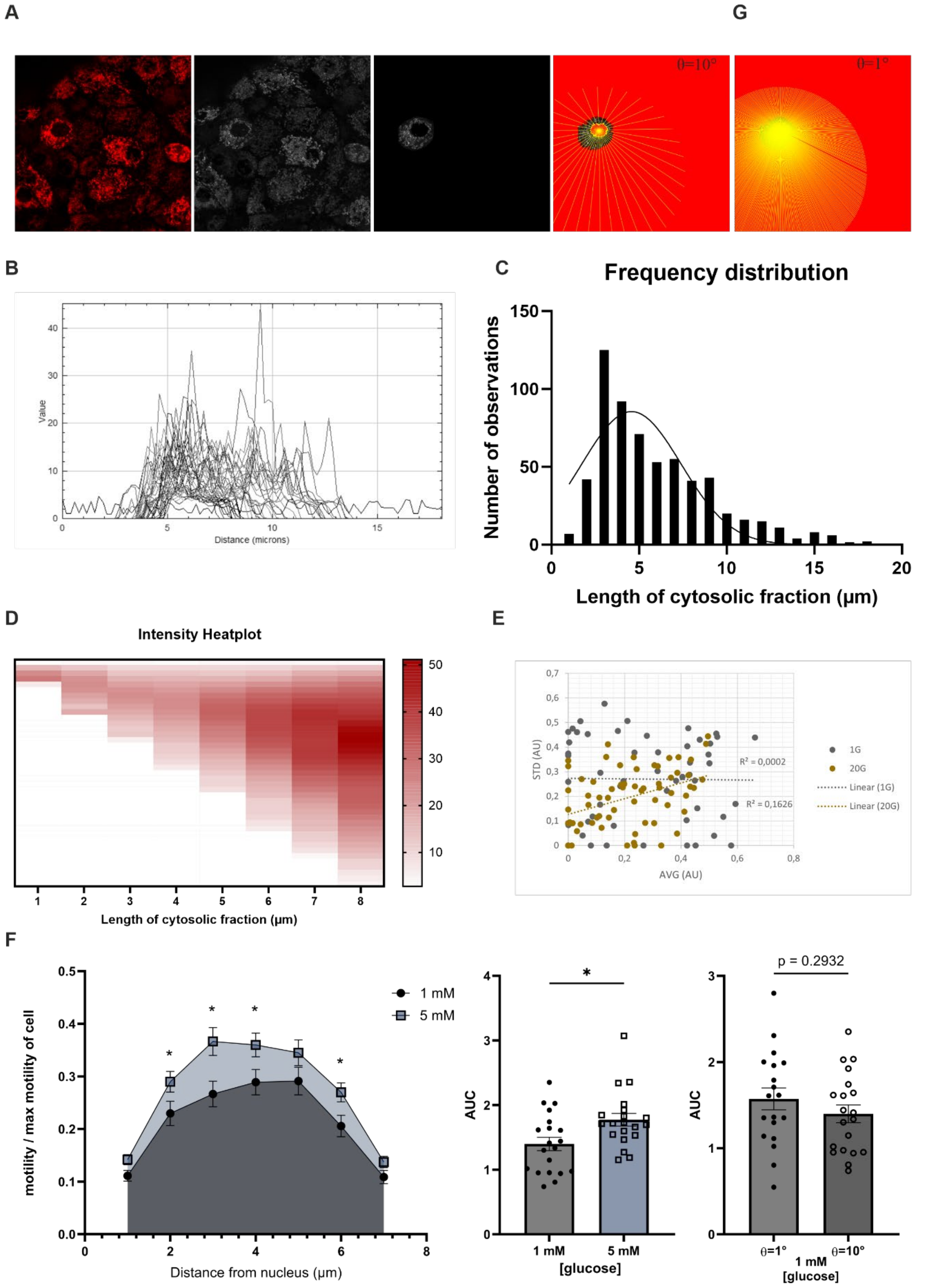

Figure S1. Establishing a method for analysis of mitochondrial motility in whole pancreatic islets ex vivo

A) Imaging analysis workflow showing a confocal image of a whole isolated islet incubated in 7 mM glucose with MitoTracker for 15 mins and a Z-project of the standard deviation of signal in each pixel (STD) from a time series of 20 images taken over 1.5 mins. Each cell is manually outlined and a threshold is applied to exclude background signal around the cell and in the nucleus. Subsequently, 36 line-ROIs spanning from the centroid of the nucleus at a 10° angle (θ=10°) from each other is obtained using original code for Fiji/ImageJ. **B)** Profile plots of STD plotted against the distance from the nucleus for each line-ROI. **C)** The frequency distribution of line-ROIs (cytosolic fractions) of different lengths fitted against the normal distribution (black line) (n=17). **D)** Intensity heat map of the average mitochondrial distribution within all cytosolic fractions (<8 μm) in a control set of islet cells incubated in 7 mM glucose (n=17). **E)** Correlation plot for STD plotted against AVG from time series of mitochondria in islet cells incubated in 1 or 20 mM glucose (n=6). **F)** Data acquired from the same raw data set as in fig. 1C but using 360 line-ROIs at a 1° angle from each other (θ=1°) (as visualised in **H**). **G)** A comparison between using θ=10° and θ=1° to analyse mitochondrial motility in 1 mM glucose.

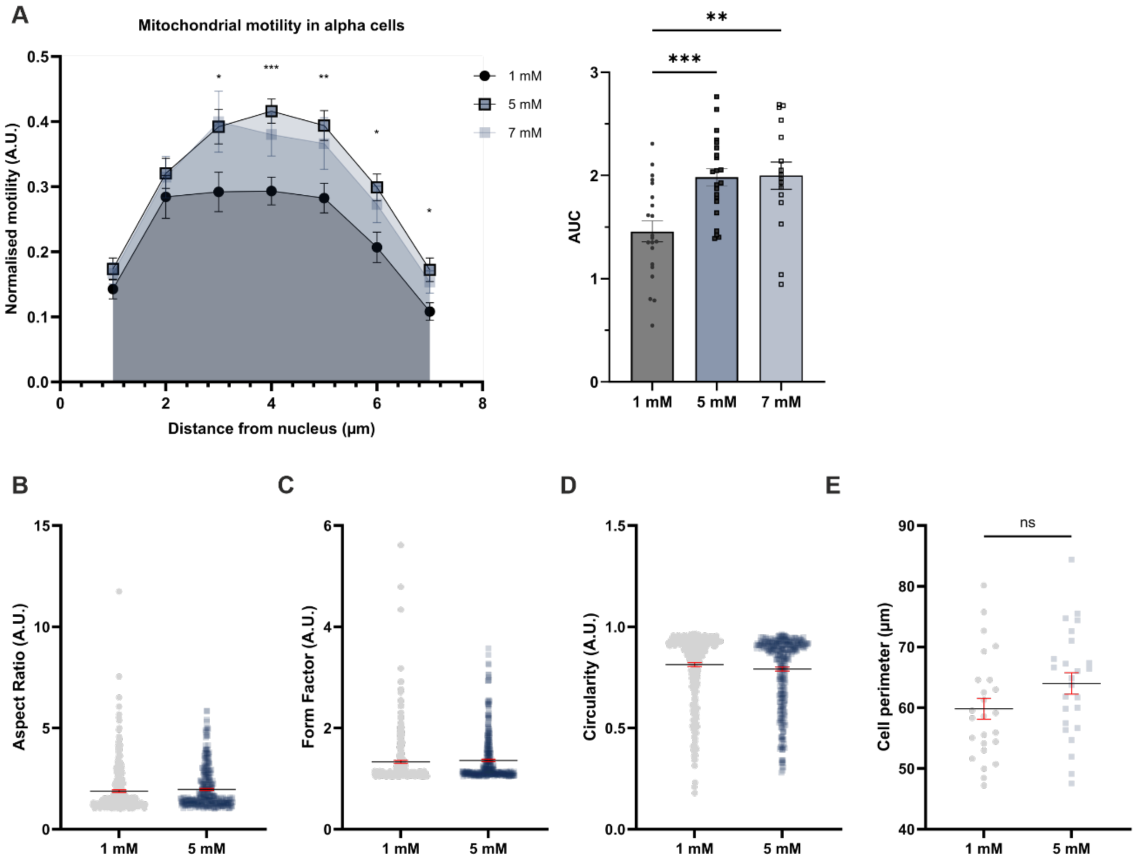

<u>Figure S2. Mitochondrial motility is comparable between 5 mM and 7 mM glucose and mitochondrial morphology and alpha cell size is unchanged after incubation in 1 mM or 5 mM glucose</u>

A) Same dataset as in figure 1E but with the addition of data from alpha cells that have been incubated in 7 mM glucose (n=16-22 alpha cells from >3 mice). **B-D)** Mitochondrial aspect

ratio, form factor, and circularity (n=309-346 mitochondria from 12-14 alpha cells). **E)** The perimeter of alpha cells incubated for 15 minutes in glucose as indicated (n=25 alpha cells from >3 independent experiments). *p<0.05, ** p<0.005, ***p<0.001 with one- or two-way ANOVA.

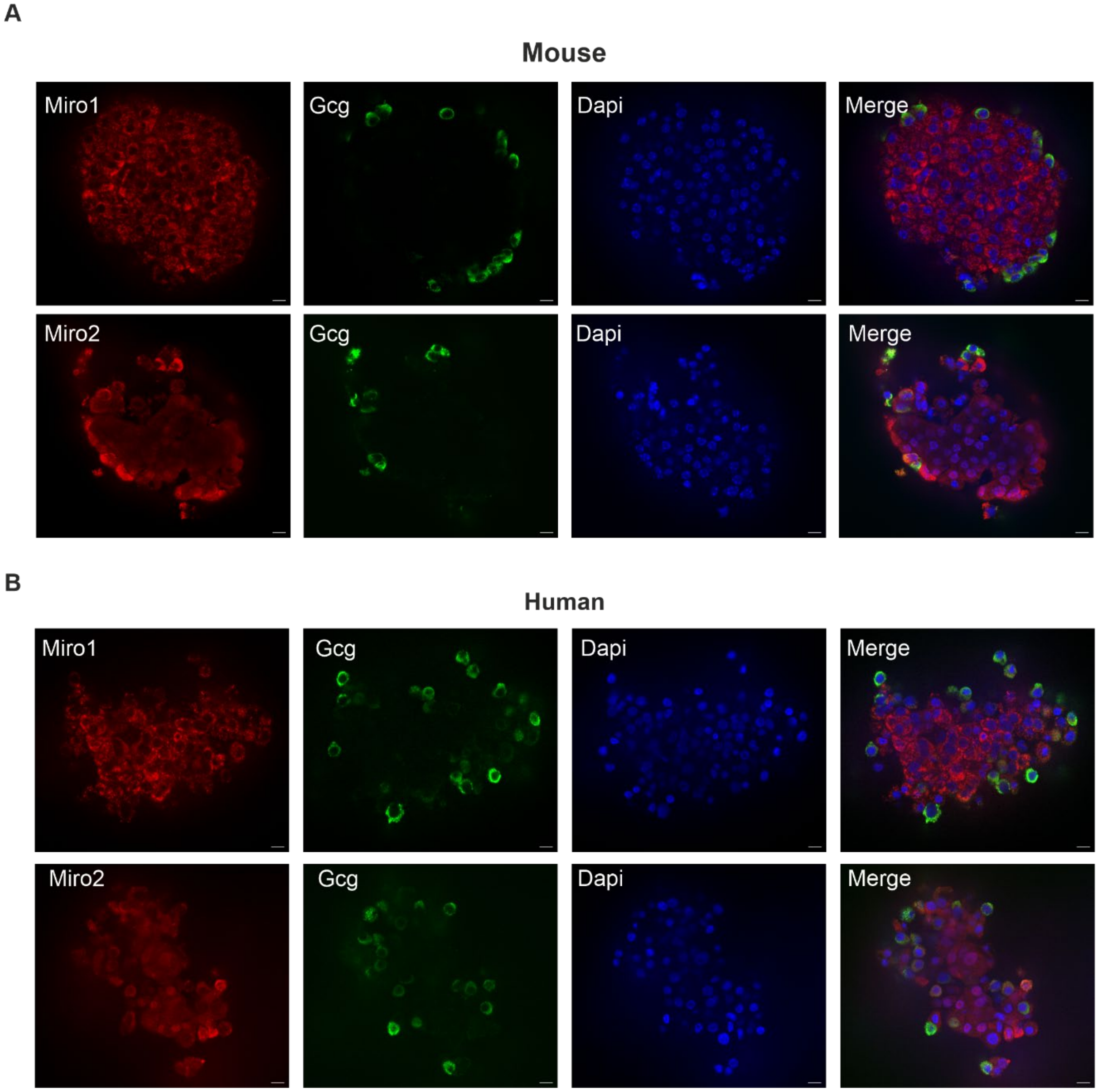

Figure S3. Miro1 and Miro2 is expressed in both alpha and beta cells in mice and humans

Immunofluorescence staining of Miro1 and -2 in whole isolated islets from **A)** mouse and **B)** human. Scalebars are 10 µm.

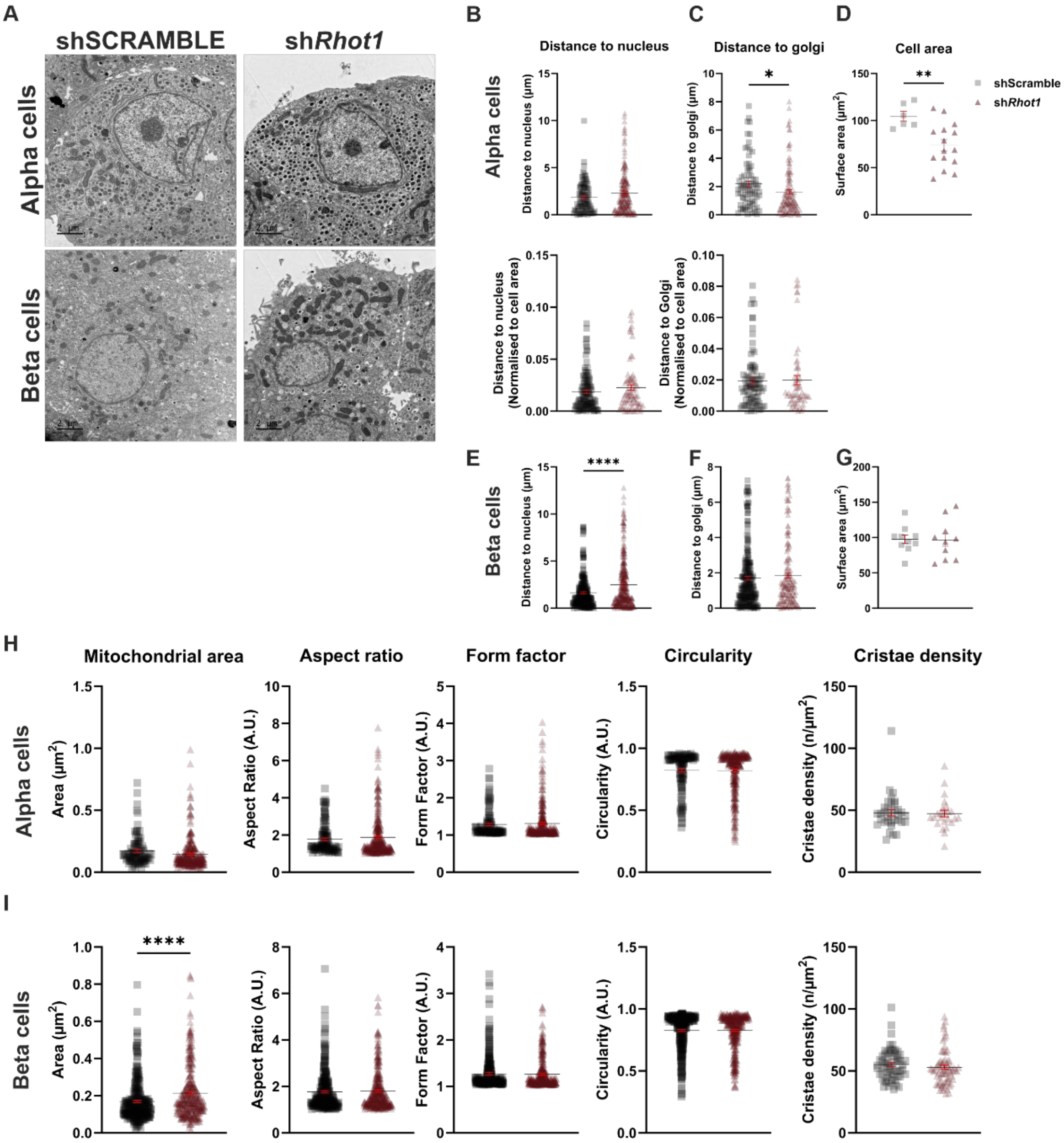

Figure S4: Reduced expression of Miro1 alters mitochondrial localisation and size in beta cells

A) Electron micrographs of alpha and beta cells infected with shSCRAMBLE or sh*RHOT1*. **B-D and E-H)** The shortest distance from each mitochondrion to the nucleus or Golgi apparatus in alpha cells (B-D) and beta cells (F-H). **D and G)** Area of alpha (D) and beta (G) cells infected with shSCRAMBLE or sh*RHOT1* (n=97-181 mitochondria from 6-15 alpha cells and 273-326 mitochondria from 10 beta cells). **H and I)** Mitochondrial area, aspect ratio, form factor, circularity and cristae density in alpha cells (J) and beta cells (K) (for cristae data: n=24-27 mitochondria from 4 alpha cells and 58-72 mitochondria from 10 beta cells). *$p<0.05$, **$p<0.005$, ****$p<0.0001$ with unpaired t-test.
